# Sex difference in alcohol withdrawal syndrome: a scoping review of clinical studies

**DOI:** 10.3389/fpsyt.2023.1266424

**Published:** 2023-09-22

**Authors:** Hayrunnisa Unlu, Marie Michele Macaron, Hande Ayraler Taner, Duygu Kaba, Burcu Akin Sari, Terry D. Schneekloth, Lorenzo Leggio, Osama A. Abulseoud

**Affiliations:** ^1^Department of Psychiatry and Psychology, Mayo Clinic Arizona, Phoenix, AZ, United States; ^2^Department of Child and Adolescent Psychiatry, Baskent University School of Medicine Hospital, Ankara, Turkey; ^3^Medical School, St George’s University of London, London, United Kingdom; ^4^Section on Clinical Psychoneuroendocrinology and Neuropsychopharmacology, Translational Addiction Medicine Branch, National Institute on Drug Abuse, and National Institute on Alcohol Abuse and Alcoholism, Baltimore, MD, United States; ^5^Department of Neuroscience, Graduate School of Biomedical Sciences, Mayo Clinic College of Medicine, Phoenix, AZ, United States

**Keywords:** sex differences, alcohol withdrawal, delirium tremens, alcohol withdrawal syndrome, gender difference, alcohol withdrawal seizures

## Abstract

**Background:**

We conducted a review of all studies comparing clinical aspects of alcohol withdrawal syndrome (AWS) between men and women.

**Methods:**

Five databases (PubMed, Cochrane, EMBASE, Scopus and Clinical Trials) were searched for clinical studies using the keywords “alcohol withdrawal syndrome” or “delirium tremens” limited to “sex” or “gender” or “sex difference” or “gender difference.” The search was conducted on May 19, 2023. Two reviewers selected studies including both male and female patients with AWS, and they compared males and females in type of AWS symptoms, clinical course, complications, and treatment outcome.

**Results:**

Thirty-five observational studies were included with a total of 318,730 participants of which 75,346 had AWS. In twenty of the studies, the number of patients presenting with or developing AWS was separated by sex, resulting in a total of 8,159 (12.5%) female patients and a total of 56,928 (87.5%) male patients. Despite inconsistent results, males were more likely than females to develop complicated AWS [delirium tremens (DT) and AW seizures, collective DT in Males vs. females: 1,792 (85.4%) vs. 307 (14.6%), and collective seizures in males vs. females: 294 (78%) vs. 82 (22%)]. The rates of ICU admissions and hospital length of stay did not show sex differences. Although variable across studies, compared to females, males received benzodiazepine treatment at higher frequency and dose. One study reported that the time from first hospitalization for AWS to death was approximately 1.5 years shorter for males and males had higher mortality rate [19.5% (197/1,016)] compared to females [16% (26/163)].

**Conclusion:**

Despite the significant heterogeneity of the studies selected and the lack of a focus on investigating potential sex differences, this review of clinical studies on AWS suggests that men and women exhibit different AWS manifestations. Large-scale studies focusing specifically on investigating sex difference in AWS are needed.

## 1. Introduction

Alcohol withdrawal (AW) is a common medical condition with characteristic clinical manifestations that take place a few hours to a few days after cessation or significant reduction in heavy and prolonged alcohol consumption. These manifestations range from mild anxiety, nausea, shaking, and agitation to seizures, delirium tremens (DT), and death (1–4). According to DSM-5 TR, less than 10% of individuals with alcohol withdrawal syndrome (AWS) will ever develop severe autonomic hyperactivity and alcohol withdrawal DT, and less than 3% of individuals will experience tonic–clonic seizures during alcohol withdrawal (5). Nonetheless, AWS is a major public health problem in the U.S. with approximately 500,000 episodes per year sufficiently severe to require pharmacological treatment (6). Approximately 50% of middle-class, highly functioning individuals with alcohol use disorder (AUD) experience AWS, and more than 80% of hospitalized AUD patients may experience alcohol withdrawal (5). Even though AWS is highly prevalent and causes significant morbidity and mortality, there is a dearth of information about its prevalence among women or sex-difference in clinical manifestations, treatment response or outcome (7, 8).

Until recently, men surpassed women by a wide margin in social and problematic alcohol drinking patterns (9, 10), and most of our clinical knowledge about AWS came from studies that enrolled predominantly (11–17) or only men (18, 19). Over the past two decades, a robust increase in alcohol consumption (20), hazardous drinking (21–23), alcohol-related emergency room visits (24), and AWS (25) have been observed among women, especially among adolescents (23, 26–29). Current management of AWS in women assumes that women exhibit clinical manifestations, respond to treatment, and develop AWS complications similar to men. However, gaining a deeper understanding of the role of sex in AWS is crucial to both recognizing and effectively managing this complex syndrome.

Animal studies have shown evidence of sex differences in AWS (30) with male rats had greater withdrawal seizure susceptibility than female rats (31, 32) and only male mice experienced increased seizure risk following repeated alcohol withdrawal episodes (33). In addition, male rats exhibited increased anxiety like behaviors during alcohol withdrawal which was demonstrated both with enhanced acoustic startle responses, elevated plus maze and suppressed social activity (32, 34, 35). One study showed a significantly lower alcohol withdrawal severity in female mice than male mice (36). Despite these well- establish sex differences in animal AWS models, there are inconsistencies between clinical AWS studies; some suggest that men tend to experience more severe AWS than women (37–41), while others demonstrate that women can also experience AWS as severe as men (42–47). It is essential to recognize the significance of sex differences in AWS, as evidenced by both animal and clinical studies, highlighting the urgency to better understand and address these differences.

To establish the findings on sex differences in AWS in the current literature and address disparities, we conducted a comprehensive scoping review of all clinical studies related to AWS. In this review, we compare outcomes including AWS symptoms, AWS complications, hospital length of stay (LOS), ICU admission rates and LOS, laboratory values, clinical course, and treatment plans between males and females. We hypothesize that women can also present with complicated AWS, because it is well-established that women are more vulnerable to developing alcohol-related complications at lower-level drinking and after a shorter duration of alcohol consumption (time from first use to dependence) compared to men (48–50). However, we expect to see that males will be more likely to suffer from alcohol withdrawal seizures, as consistently shown in animal studies.

## 2. Methods

We conducted a scoping review of the literature by searching five databases, PubMed, Cochrane, EMBASE, Scopus and Clinical Trials. The search strategy was designed and conducted by an experienced librarian. Controlled vocabulary with keywords was used to search for studies describing alcohol withdrawal and sex difference. Supplementary file 1 provides an outline the search strategy listing all the search terms used and how they are combined. This review was registered prospectively with PROSPERO (CRD42023394108).

### 2.1. Article selection and quality assessment

**Figure 1:**
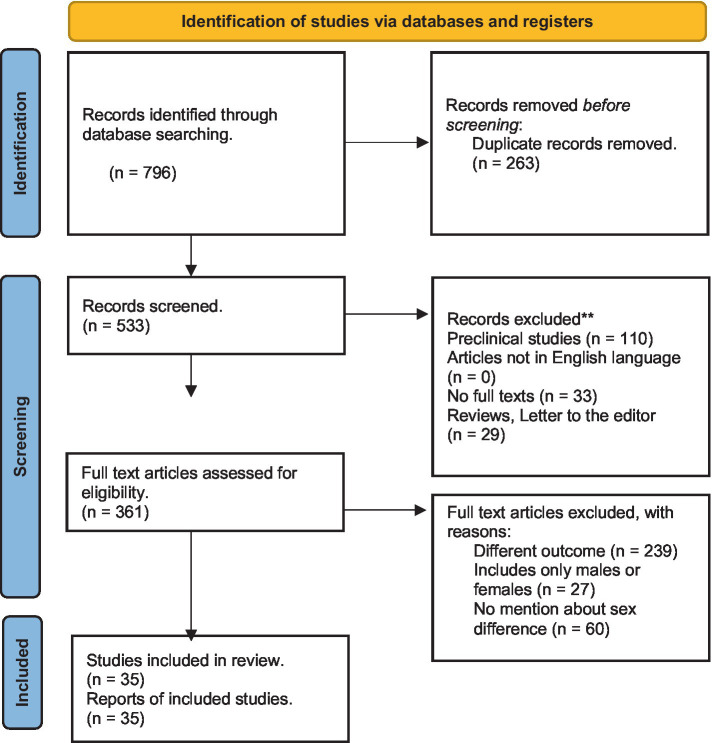
PRISMA flowchart for article selection.

The search was conducted on May 19, 2023, and yielded 796 records. An abstract review identified articles addressing clinical symptoms and their management; these were shortlisted. From all original articles (open-label or double-blind trials, prospective or retrospective observational studies, and cohort or cross-sectional studies) written in English, we included those that met the following criteria: (1) study participants consisted of both male and female patients experiencing alcohol withdrawal syndrome, (2) results compared males and females in at least one of the following outcomes: (i) AWS symptoms, (ii) AWS complications (i.e., delirium tremens, seizures, hallucinations, or mortality), (iii) ICU admission rates, (iv) hospital length of stay, (v) laboratory values and clinical course, or (vi) treatment plan. We excluded pre-clinical studies, articles not in English language, articles not in full text, studies that do not compare at least one outcome of interest between males and females, reviews, commentaries, or letters to the editor. All authors agreed on the inclusion and exclusion criteria. The inclusion or exclusion of individual studies was discussed between the two lead authors (HU and MMM). Articles with non-agreement were discussed with the senior author (OAA). The full texts of the shortlisted articles were reviewed. Cross-references were searched from selected studies and relevant articles were also evaluated for inclusion (Figure 1).

The quality of each study was independently evaluated by the two lead authors (HU and MMM). Cohort studies were assessed using the Newcastle Ottawa Assessment Scale (NOAS) (51). This scale evaluates various aspects, including sample selection (representativeness of the target population, sample size, comparability between respondents and non-respondents, and outcome ascertainment), comparability (comparability between subjects in different outcome groups), and outcomes (method of outcome measurement and statistical tests employed). A maximum of four stars can be given to a study under the category of selection, two stars under the comparability category, and three stars for the outcome category. The adapted for cross sectional studies NOAS was used to assess cross sectional studies (52). This scale evaluates sample selection (representativeness of the cases, sample size, non-response rate, and ascertainment of screening/surveillance tool), comparability (potential confounders), and outcome (outcome assessment, and statistical test). Sample selection can be awarded a maximum of 5 stars, comparability can be awarded a maximum of one star, and outcome can be awarded a maximum of 3 stars. Finally, observational studies were assessed based on the observational studies NOAS (53). This scale evaluates sample selection (representativeness of the exposed cohort, selection of the non-exposed cohort), exposure (ascertainment of exposure, exposure dose, retrospective/prospective dose ascertainment), comparability (confounding), outcome assessment, and follow up (period and adequacy). Sample selection and follow-up assessment can be awarded a maximum of two stars; comparability and outcome assessment can be rewarded a maximum of one star each; ascertainment of exposure can be awarded 4 stars maximum. Discrepancies in quality assessment were resolved through discussions involving a senior author (OAA) until a consensus was reached. The results of the quality assessment for all included studies can be found in Supplementary file 2. The data were synthesized, and the relevant findings are discussed below.

### 2.2. AWS severity assessment scales

Among studies included, three studied used the Clinical Institute Withdrawal Assessment of Alcohol Scale, revised (CIWA-Ar) (54–56) to measure the severity of AWS. Additionally, two studies used the Clinical Institute Withdrawal Assessment of Alcohol (CIWA-A) (57, 58), and one used the Alcohol Withdrawal Scale (AWS) (59).

#### 2.2.1. Clinical institute withdrawal assessment of alcohol scale, revised

The CIWA-Ar scale is a 10-item survey that assesses a patient’s symptoms and scores a patient’s severity of symptoms. Scores on the CIWA-Ar range from 0 to 67 points. The CIWA-Ar evaluates the following signs and symptoms: (1) nausea and vomiting, (2) tremors, (3) sweating; (4) anxiety; (5) agitation; (6) tactile disturbances, (7) auditory disturbances, (8) visual disturbances, (9) headache; and (10) disorientation or clouding of sensorium. Each item is scored 0–7 except item 10, which is scored 0–4 (60).

#### 2.2.2. Clinical institute withdrawal assessment for alcohol

The CIWA-A scale is a 15-item survey that assesses a patient’s symptoms and scores a patient’s severity of symptoms. Scores on the CIWA-A range from 0 to 86 points. The CIWA-A scale quantifies the following signs and symptoms: (1) nausea and vomiting, (2) tremors, (3) sweating, (4) tactile disturbances, (5) auditory disturbances, (6) visual disturbances, (7) hallucinations, (8) clouding of sensorium, (9) quality of contact, (10) anxiety, (11) agitation, (12) thought disturbances, (13) convulsions, (14) headache, and (15) flushing. Items 1, 2, 3, 9, 10, 11, 13, and 14 are scored 0–7, while items 4, 5, and 6 are scored 0–6, items 7, and 12 are scored 0–3, item 8 is scored 0–4 and item 15 is scored 0–2 (61).

#### 2.2.3. Alcohol withdrawal scale

The AWS scale is an 11-item survey that assessing both somatic symptoms and mental symptoms and scores range from 0 to 68 points. AWS somatic symptoms include (1) Pulse rate (per min), (2) Diastolic blood pressure (mmHg), (3) Temperature, (4) Breathing rate (per min), (5) Sweating, and (6) Tremor. Items scores range from 0 to 3 except for item 4, which is scored 0–2. AWS mental symptoms include: (1) Agitation, (2) Contact, (3) Orientation (time, place. Person, situation), (4) Hallucinations (optical, acoustic, and tactile), and (5) Anxiety. Each item is scored 0–4, except for item 2, which is scored 0–3, and item 5, which is scored 0–2. AWS scale gives a total score by combining these two sub scores (somatic + mental). AWS Scale scores were categorized as <5 mild, 6–9 moderate, ≥10 severe withdrawal (62).

## 3. Results

Thirty-five observational studies met the inclusion/exclusion criteria. Almost half of the included studies were conducted in the U.S. (*n* = 15), followed by Spain (*n* = 8), Germany (*n* = 6), including a study conducted in both Germany and Nigeria, and Poland (*n* = 2). The other 4 studies came from Denmark, Croatia, Sweden, and Australia. More than a third (*n* = 13) were published prior to the end of 2000. The details of these studies are summarized in Table 1. These 35 studies included a total of 318,730 participants of which 75,346 presented with or developed AWS. Twenty of the studies (*n* = 65,087) separated the number of AWS patients by sex, resulting in a total of 8,159 (12.5%) female patients and a total of 56,928 (87.5%) male patients.

**Table 1:** Study characteristics.

Citation	Timeline	Country	Study design	Setting	Study population	Total number of patients	Total number of female patients	Total number of male patients	Age of the population	Number of patients that developed/presented with AWS	Number of female patients that developed/presented with AWS	Number of male patients that developed AWS	*p*-value for sex difference in number of AWS
Amaducci et al. (63)	October 1, 2019–August 31, 2020	USA	Retrospective observational	Tertiary Care Hospital	Patients presenting with AWS	324	80	244	*N* = 166 between age 41–60 *N* = 94 between age 21–40 *N* = 64 age > 60	324–entire population	80	244	NR
Barrio et al. (64)	1988–1993	Spain	Prospective observational	Internal medicine department	Admitted heavy drinkers	256	76	180	Mean [R] = 42 [19–75]	150	40	110	*p* = 0.2
Berggren et al. (65)	1997–1998	Sweden	Retrospective cohort	Alcohol treatment unit	Patients with alcohol dependence and AWS	314	57	277	Mean (SD) = 49 (10)	334–entire population	57	277	NR
Campos et al. (2)	1996–2006	Spain	Retrospective cohort	University hospital	Admitted patients with final diagnosis of AWS	1,265	180	1,085	Mean [*R*] = 49 [18–89]	1,265–entire population	180	1,085	NR
Canales et al. (42)	2010–2014	USA	Retrospective observational	Public hospital	Patients with discharge diagnosis of AWS	1,496	118	1,378	NR	1,496–entire population	118	1,378	*p* < 0.001
Deshmukh et al. (66)	NR	USA	Prospective observational	Community treatment programs or from a veterans administration medical center	AUD patients	128	62	66	NR	68	27	41	*p* < 0.025
Eyer et al. (44)	2000 and 2009	Germany	Retrospective cohort	Hospital	Inpatients with severe AWS	827	221	606	Seizure patients [Mean (SD)] =44 (10) No-seizure patients = 45 (10)	827–entire population	221	606	NR
Foy et al. (58)	1987–1993	Australia	Prospective cohort	General hospital	AUD patients	539	102	437	Complicated AWS = 53 [*R* = 23–87] Non-complicated AWS =51 [*R* = 19–88]	539–entire population	102	437	NR
Gómez-Méndez et al. (67)	1999–2010	Spain	Retrospective cohort	Hospital	AWS patients	56,395	6,749	49,646	Mean (SD) = 50,9 (12.5)	56,395–entire population	6,749	49,646	NR
Isichei et al. (68)	12 months but NR	Nigeria, Germany	Prospective observational	Hospital	AUD patients	202	49	153	Mean [*R*] = 27 [15–59] years in Nigeria, 31 [15–63] in Germany	NR	NR	NR	NR
Himmelstein (69)	January 1, 1978–June 30, 1978	USA	Retrospective cohort	Urban public hospital	Patients with alcohol related diagnoses	2,036	385	1,651	NR	281	17	264	*p* < 0.05
Jarque-Lopez et al. (37)	1998–1999	Spain	Prospective cohort	Emergency room	Admitted heavy drinkers	278	23	224	NR	NR	NR	NR	NR
Lewis et al. (41)	1967–1968	USA	Prospective cohort	Psychiatric hospital in St louis	Admitted patients with alcoholism	259	103	156	NR	NR	NR	NR	NR
Marchand et al. (55)	January 2013 through December 2016	USA	Retrospective cohort	Trauma center in northeast Ohio	Admitted trauma patients	1,011	269	742	Mean (SD) = 47.2 (18.1)	42	6	36	NR
Martins et al. (56)	N/A	USA	Prospective observational	Outpatient treatment	Treatment-seeking adults with current DSM-5 AUD	80	31	49	Mean (SD) = 36.6 (11.24)	80–entire population	31	49	NR
Monte et al. (45)	1987 and 2003	Spain	Retrospective cohort	Hospital (medical or surgical services)	Admitted AWS patients	303	36	267	Mean (SD) = 45 (12)	303–entire population	36	267	NR
Monte et al. (70)	1987 and 2003	Spain	Retrospective cohort	Hospital (medical or surgical services)	Admitted AWS patients	436 patients, 539 hospitalizations	47 hospitalizations	492 hospitalizations	Mean (SD) = 45 (12)	436–entire population	NR	NR	NR
Monte-Secades et al. (46)	January 1, 2013–December 31, 2014	Spain	Prospective observational	Hospital	AWS patients	219	12	207	Mean (SD) = 54.4 (11.5)	219–entire population	NR	NR	NR
Nedic Erjavec et al. (38)		Croatia	Cross-Sectional	Hospital	Medication-free alcohol dependent patients	661	123	538	Median (IQR) = 49 (42. 55) for smokers 52 (44,60) for non-smokers	153	NR	NR	NR
O’Connor et al. (47)	NR	USA	Retrospective cohort	Outpatient	Outpatient AWS patients seeking treatment	179	34	145	NR	179–entire population	34	145	NR
Ring et al. (71)	2019	Poland	Retrospective cohort	Hospital	AWS patients	656	95	561	Mean (SD) = 45.51 (11.83)	506	69	437	NR
Salottolo et al. (54)	2010–2014.	USA	Retrospective cohort	Three US trauma centers	Trauma patients	28,101	11,756	16,345	*N* = 14,057 age ≥ 55	246	41	205	NR
Sanvisens et al. (8)	2014 and 2016	Spain	Cross-sectional	Outpatient clinics at hospitals	AUD patients	313	79	234	Mean [*R*] = 50 years [43–54]	230	51	179	NR
Schimmel et al. (25)	1 March 2019–31 May 2020	USA	Retrospective cohort study	Emergency department	Patients with alcohol related presentations	4,583	1,021	3,562	Median = 46 (in 2019) 47 (in 2020)	375	NR	NR	NR
Schuckit et al. (39)	February 1991–March 1994	USA	Cross-Sectional	Research centers	AWS patients	1,648	540	1,108	Mean (SD) = 37.9 (12.54)	NR	NR	NR	NR
Sørensen et al. (40)	1994–2005	Denmark	Prospective cohort	Alcohol treatment facilities	AUD patients	3,582	1,035	2,547	Mean [*R*] = 45 [19–82]	NR	NR	NR	NR
Soyka et al. (72)	1980–1985	Germany	Retrospective cohort	Hospital	Patients with acute alcohol psychosis	154	33	121	AW delirium patients (Median) = 38.5 Hallucinative patients = 39.3	103	22	81	NR
Soyka et al. (73)	NR	Germany	Retrospective cohort	Hospital	AUD patients	906	325	581	NR	NR	NR	NR	NR
Soyka et al. (57)	January 2004 and March 2005	Germany	Retrospective cohort	Hospital	AUD patients diagnosed with AWS	540	115	425	Mean (SD) = 45.7 (9.4)	540–entire population	115	425	NR
Steel et al. (74)	October 1, 2012–September 30, 2013	USA	Retrospective cohort	Hospital	Medical inpatients with AWS	209,151	8,782	200,369	Mean (SD) = 67.9 (12.8)	9,727	NR	NR	NR
Stewart and Brown (75)	NR	USA	Cross-sectional	Inpatient substance abuse treatment program	Adolescents seeking AUD treatment	166	67	99	Mean (SD) = 16.02 (1.27)	8	NR	NR	NR
Tavel et al. (76)	January 1 1950–December 31 1958	USA	Retrospective cohort study	Hospital	Patients with DT	330 DT cases	45 DT cases	285 DT cases	Mean (SD) = 42.5 (10.4)	NR	NR	NR	NR
Wetterling and Junghanns (59)	NR	Germany	Prospective cohort	Hospital	AUD patients	110	35	75	Mean (SD) = 44.7 (10.5)	63	NR	NR	NR
Wojnar et al. (43)	1973–1987	Poland	Retrospective cohort	Hospital	Admitted AWS patients	1,179	163	1,016	Mean (SD) = 39.95 (9.98)	1,179–entire population	163	1,016	NR
Worner and Lechtenberg (77)	November 1987–December 1989	USA	Cross-sectional	Detoxification unit	AUD patients	400	60	340	Mean (SD) = 41.4 (10.5)	NR	NR	NR	NR

SD, Standard Deviation; R, Range; IQR, Interquartile Range.

NR, Not reported.

NS, Non significant.

### 3.1. Sex difference in AWS prevalence

Gómez-Méndez et al. (67) reported that among patients admitted with AWS, whether primary (as reason for admission) or secondary diagnosis of AWS, 88% (49,646/56,393) of them were males. Sanvisens et al. (8) conducted a study with patients who had requested treatment for AUD and found that males [76.5% (179/234)] experience AWS more than females [64.6% (51/79), *p* = 0.038]. Salottolo et al. (54) reported that among 28,101 patients [57.94% (16,140/28,101) males, 42.06% (11,715/28,101) females] admitted to trauma centers, patients who developed AWS were mostly males [205/246 (83.33%), *p* < 0.001]. Himmelstein’s study showed a significantly higher rate of AWS in men (264/281 patients, *p* < 0.05) (78). Marchand et al.’s study (55) showed a non-significant trend for male sex as a risk factor for AWS [Relative risk (RR) = 2.2, 95% CI: 0.9–5.1, *p* = 0.06]. Steel et al.’s study with inpatient veterans showed that male sex was associated with an increased probability of inpatient AWS (*p* < 0.001) (74). Among a cohort of males and females with alcohol dependence, Deshmukh et al. (66) observed higher endorsement of AW criterion among men (*p* < 0.025). Similarly, Jarque-López et al. reported major AWS was more common among men (224/278, *p* < 0.001) (37). Interestingly, one study in adolescents reported that the mean number of AWS symptoms (i.e., irritability, tremor, nausea and/or vomiting) was higher among females [Mean (SD) for males vs. females = 9.31 (5.6) vs. 11.1 (5.7), *p* < 0.05] (75).

Nedic Erjavec et al. (38) showed a non-significant trend toward higher prevalence of withdrawal symptoms in males [males vs. females: 24% (129/538) vs. 17% (21/123), *p* = 0.08]. Sex was not found to be associated with alcohol related hospital visits, including AWS and its complications, in Schimmel et al.’s study [female Odds Ratio (OR) of alcohol withdrawal 0.86 (0.66–1.14) and withdrawal complications 0.87 (0.67–1.12)] (25).

### 3.2. Sex difference in clinical characteristics

Wojnar et al. (43) reported that women with AWS were non-significantly older [males (*n* = 1,016) vs. females (*n* = 163): 41.2 ± 11 vs. 39.8 ± 9.8 years] and drank significantly less alcohol than men (*p* < 0.0001). Also, the time from intensive drinking to the first withdrawal episode was 5 years shorter in women (*p* < 0.0001). Of note, this study did not define intensive drinking. O’Connor et al. (47) reported no significant difference in age [males (n = 145) vs. females (n = 34): 38 ± 10 vs. 36 ± 10 years] or in the amount of alcohol intake. In their study, the duration of alcohol misuse before developing AWS was found to be 3 years less in women than in men, though the finding was not significant (19.1 vs. 16.1 years, *p* = 0.08). Canales et al. (42) recently reported that among patients with AWS, women were younger than men [males (n = 1,372) vs. females (n = 118): 45.6 vs. 43.9 years, *p* = 0.009]. Schuckit et al. (39) compared 160 men and 51 women with severe AWS, the two groups were not significantly different in the age of onset of alcohol dependence (males vs. females: 24.5 ± 8.9 vs. 23.5 ± 8.8 years), years of heavy drinking (14.2 ± 10.0 vs. 11.0 ± 8.5), frequency of drinking (6.0 ± 1.8 vs. 5.4 ± 2.1 days per week), and total number of alcohol use problems (33.3 ± 6.2 vs. 32.5 ± 6.2). Family history of AUD was found to be a risk factor for AWS in women (OR = 2.85, 95% CI: 1.07–7.54) (8) while amount of alcohol consumption was a risk factor for AWS in both men and women, although it was more likely in women (75). Earlier onset of alcohol consumption (OR for every 5 years = 1.89, 95% CI:1.69–2.08) was associated with increased probability of AWS in men (8).

### 3.3. Sex difference in AWS medical and psychiatric comorbidities

Wojnar et al. found that personality, anxiety, and depressive disorders, benzodiazepine and barbiturate misuse were more common among women with AWS (*p* < 0.0001) (43). In contrast, Schuckit et al. (39) reported that there was no sex difference regarding a number of different substances used (males vs. females: 2.1 ± 1.9 vs. 2.4 ± 1.8), the pattern of exposure to various drugs, history of medical conditions, and psychiatric symptoms. Women tended to report more past depression (females vs. males: 94% vs. 81%); however, there was no statistically significant difference after Bonferroni corrections. Comorbid cannabis use (OR = 2.8, 95% CI:1.04–7.7) was associated with increased probability of AWS in men (8).

Canales et al. (42) reported significantly more females had pancreatitis compared to males [females vs. males: 21% (4/19) vs. 6% (13/220) *p* = 0.03], while O’Connor et al. (47) found no significant sex difference [males vs. females: 6% (6/144) vs. 4% (2/34), P = ns]. In Canales’s study, 32% (6/19) of females and 19% (42/220) of males (*p* = 0.2) admitted to the medical intensive care unit had elevated liver enzymes, while 41% (90/220) of males and 21% (4/19) of females had pneumonia (*p* = 0.06), and 5% (12/220) of males and none of the females (0/19) developed sepsis (*p* = 0.06) (42). Ring et al. (71) evaluated comorbid general medical conditions in AWS patients and concluded that cardiovascular system diseases were the most common medical comorbidity in both males 80.6% (100/124) and females 65.4% (17/26) with AWS. Men aged 50–59 years with DT had significantly more pneumonia than men without DT (*p* = 0.007), while no similar difference was found among women (71).

Only two studies reported sex differences in laboratory values at time of admission for AWS. Women with AWS were found to have significantly more anemia [females vs. males: 39.6% (101/255) vs. 16.2% (312/1931), *p* < 0.0001] and hypokalemia [38% (97/255) vs. 21.1% (408/1931), *p* < 0.0001], while men had more hypoproteinemia [males vs. females: 3.4% (65/1,931) vs. 2.7% (7/255), *p* < 0.01], higher liver enzyme alanine aminotransferase (ALT) [68.5% (1,322/1,931) vs. 60% (153/255), *p* < 0.05] (43) and more frequent thrombocytopenia defined as platelet count less than 150,000/L; [37% (102/277) vs. 12% (7/57), *p* < 0.001] (65).

### 3.4. Sex difference in AWS symptoms, withdrawal scores, hospital length of stay and rate of ICU admissions

Deshmukh et al. (66) observed that men experienced significantly more anxiety [males vs. females: 89.7% (35/39) vs. 61.3% (19/31), *p* = 0.011] and a non-significant trend toward more tremors (*p* = 0.08). Wetterling and Junghans (59) did not observe sex differences in peak AWS scores (6.8 ± 3.9 vs. 6.1 ± 3.3, P = ns). O’Connor et al. (47) did not observe sex differences between men and women in AWS symptoms [(frequency of tremor, seizures, level of consciousness = 89% (129/145) vs. 91% (31/34), (P = ns), frequency of anxiety, agitation, hallucinations = 94% (136/145) vs. 97% (33/34), P = ns)] and its severity. Martins et al. (56) showed that the mean CIWA-Ar score was not significantly different between males (*n* = 49) and females (*n* = 31): (4.9 ± 3.8 vs. 4.1 ± 3.8, alfa = 0.7). Wojnar et al. (43) reported that women with AWS required a longer course of hospital stay (14.6 ± 10.6 vs. 10.6 ± 8.4 days, *p* < 0.0001), while Canales et al. (42) reported no significant differences between males and females in hospital LOS (7.3 ± 5.0 vs. 9.4 ± 6.1 days, *p* = 0.1), rate of ICU admission [16% (220/1,378) vs. 16% (19/118), or ICU LOS (4.0 ± 4.9 vs. 5.2 ± 4.8 days, *p* = 0.06)]. Ring et al. (71) concluded that there was no sex difference in hospital LOS due to AWS and DT (*p* = 0.3).

### 3.5. Sex differences in delirium tremens and AW seizures

Eleven studies (total *n* = 9,071: male = 7,147, females = 1,924) separated the number of DT patients by sex, resulting in a total of 1,792 (85.4%) males and a total of 307 (14.6%) females. 25% (1,792/7,147) of males and 17% (307/1,924) of females experienced DT (37, 38, 40–45, 47, 54, 68).

Seven studies (total *n* = 4,940: male = 3,974, females = 966) separated the number of patients with AW seizures by sex, resulting in a total of 294 (78%) males and a total of 82 (22%) females. 7.4% (294/3,974) of males and 8.5% (82/966) of females experienced AW seizure (42–44, 47, 73, 77, 78). Three studies merged AWS complications (DT, hallucinations, seizures), with a total of 17.5% of males (330/1,876) and 10% of females (69/689) in these three studies developed complicated AWS (39, 46, 78). Wojnar et al. (43) found that women were more likely than men to present with DT upon admission [males vs. females: 53.5% (996/1,862) vs. 63.6% (152/239), *p* < 0.01], while the frequency of AW seizures was higher in men [2.8% (52/1,862) vs. 0.5% (1/239), *p* < 0.001]. Lewis et al. (41) found that men experienced significantly more DT than women [35.5% (55/156) vs. 17.5% (18/103), *p* < 0.002]. Sorensen et al. also found that the risk of DT was significantly higher in men [11.9% (303/2,547) vs. 7.9% (82/1,035), *p* < 0.001]. In fact, male sex was shown to be the strongest predictor of DT incidence [Hazard Ratio (HR) = 1.62 (95% CI, 1.25–2.08] (40). Jarque-Lopez et al. (37) reported that 39% (87/224) of men but none of the women (0/23) experience DT (*p* < 0.001). Nedic Erjavec et al. (38) found a significant sex difference in the frequency of DT [8% (43/538) vs. 2% (3/123), *p* = 0.027], however; withdrawal symptoms did not differ significantly between the sexes (*p* = 0.08). In Himmelstein’s study (78), there was significant sex difference, and only 6% (17/281) of AWS and DT patients were women (*p* < 0.05). This study reported that 7.7% (6/78) of DT and hallucinations patients and 22.5% (9/40) of seizure patients were women. Schuckit et al. (39) concluded that patients with more severe AWS (history of DTs and/or convulsions) tended to be males (75.8% of severe AWS patients, *p* < 0.01). The number of withdrawal symptoms in the worst episode (5.9 ± 2.4 vs. 6.1 ± 2.1) and the number of days of the longest withdrawal episode (5.6 ± 4.6 vs. 4.8 ± 4.7) were not found to be significantly different between males and females. Soyka et al. (57) found that AWS complications [DT (*p* < 0.01), seizures (*p* = 0.01), and hallucinations (*p* < 0.001)] were more common among men. A previous study by Soyka et al. (57) reported that male to female ratio was 3.7:1 for DT [78.6% (*n* = 81) vs. 21.4% (*n* = 22)] and 3.6:1 for hallucinations [(78.4% (*n* = 40) vs. 21.6% (*n* = 11)] (72). In Tavel et al.’s study (76), the entire population of the study consisted of DT patients and 86% were males (285/330).

In contrast, Canales et al. (42) concluded that there was no significant sex difference regarding AWS complications, 32% (6/19) of women and 22% (48/220) of men in medical intensive care unit (MICU) developed seizures (*p* = 0.32), while 37% (82/220) of men and 26% (5/19) of women developed delirium (*p* = 0.7). O’Connor et al.’s results supported no significant sex difference in DT [males vs. females: 12% (17/144) vs. 9% (3/34), P = ns] and AW seizures [20% (29/144) vs. 9% (3/34), P = ns] (47). Eyer et al. (44) reported no significant sex difference among patients with DT [males vs. females: 72% (33/46) vs. 28% (13/46), *p* = 0.8] and AW seizures [72% (44/61) vs. 28% (17/61), *p* = 0.8]. Monte et al. (45) and Monte-Secades et al. (46) did not find significant sex differences in complicated AWS [Monte Secades et al. study: males vs. females: 47% (98/207) vs. 57% (12/21), *p* = 0.3], Monte et al. study: [males vs. females: 26% (128/492) vs. 40% (19/47), *p* = 0.5].

Similarly, Worner and Lechtenberg (77) reported that 27% of females (15/56) and 21% of males (69/321) experienced AW seizures. Soyka et al. (73), found no sex difference in prevalence of AW seizure [15.5% (90/581) vs. 14.2% (46/325), P = ns]. Ring et al. reported that 17.3% (26/150) of DT patients were women, however they concluded that sex was not a significant predictor of developing DT (*p* = 0.46) (71). Barrio et al. (64) observed no sex difference in complicated AWS [61.1% (110/180) vs. 52.6% (40/76), *p* = 0.2]. Foy et al. (58) reported that 22.5% (23/102) of females and 21% (90/437) of males developed AW complications (e.g., seizures, hallucinations, DT).

Salottolo et al. (54) evaluated trauma patients for the development of AWS and its complications; 83% (205/246) of AWS patients and 85% (23/27) of DT patients were males.

Isichei et al. (68) compared the incidence of DT between two samples from Nigeria and Germany. DT [6% (6/101)] and hallucinations [3% (3/101)] were observed only in males in Nigerian cohort. In the German cohort, 24% (8/37) of females and 29% (19/64) of males experienced DT while 16% (6/37) of females and 9% (6/64) of males experienced hallucinations (Table 2).

**Table 2:** AWS complications.

Study	Number of female DT patients/total number of female patients (percentage)	Percentage of female DT patients from the total number of DT patients	Number of male DT patients/total number of male patients (percentage)	Percentage of male DT patients from the total number of DT patients	*p*-value for sex difference in DT	Number of female AW seizure patients/total number of female patients (percentage)	Percentage of female AW seizure patients from the total number of AW seizure patients	Number of male AW seizure patients/total number of female patients (percentage)	Percentage of male AW seizure patients from the total number of AW seizure patients	*p*-value for sex difference in seizures	Number of female hallucination patients/total number of female patients (percentage)	Percentage of female hallucination patients from the total number of hallucination patients	Number of male hallucination patients/total number of female patients (percentage)	Percentage of male hallucination patients from the total number of hallucination patients	*p*-value for sex difference in hallucinations
Wojnar et al. (43)	152/239 (63.6%)	13.25%	996/1862 (53.5%)	86.75%	*p* < 0.01	1/239 (0.5%)	2%	52/1862 (2.8%)	98%	*p* < 0.001	NR	NR	NR	NR	NR
Monte et al. (45)	19/47 (40%)	13%	128/492 (26%)	87%	NR	NR	NR	NR	NR	NR	NR	NR	NR	NR	NR
Monte-Secades et al. (46)	12/21 (57%) DT or seizure	10.9% DT or seizure	98/207 (47%) DT or seizure	89.1% DT or seizure	NR	12/21 (57%) DT or seizure	10.9% DT or seizure	98/207 (47%) DT or seizure	89.1% DT or seizure	NR	NR	NR	NR	NR	NR
Worner and Lechtenberg (77)	NR	NR	NR	NR	NR	15/56 (%27)	18%	69/321 (%21)	82%	NR	NR	NR	NR	NR	NR
Lewis et al. (41)	18/103 (17.5%)	25%	55/156 (35.3%)	75%	*p* < 0.002	NR	NR	NR	NR	NR	NR	NR	NR	NR	NR
Sørensen et al. (40)	82/1035 (7.9%)	21.30%	303/2547 (11.9%)	78.70%	*p* < 0.001	NR	NR	NR	NR	NR	NR	NR	NR	NR	NR
Jarque-Lopez et al. (37)	0/23 (0%)	0%	87/224 (39%)	100%	*p* < 0.001	NR	NR	NR	NR	NR	NR	NR	NR	NR	NR
Nedic Erjavec et al. (38)	3/123 (2%)	6.50%	43/538 (8%)	93.50%	*p* = 0.027	NR	NR	NR	NR	NR	NR	NR	NR	NR	NR
Himmelstein et al. (69)	6/128 (4.7%) DT and hallucinations	7.7% DT and hallucinations	72/561 (12.8%) DT and hallucinations	92.3% DT and hallucinations	NR	9/128 (7%)	22.50%	31/561 (5.5%)	77.50%	NR	6/128 (4.7%) DT and hallucinations	7.7% DT and hallucinations	72/561 (12.8%) DT and hallucinations	92.3% DT and hallucinations	NR
Schuckit et al. (39)	51/540 (9%) DT and/or seizure	24.2% DT and/or seizure	160/1108 (14%) DT and/or seizure	75.8% DT and/or seizure	NR	51/540 (9%) DT and/or seizure	24.2% DT and/or seizure	160/1,108 (14%) DT and/or seizure	75.8% DT and/or seizure	NR	NR	NR	NR	NR	NR
Soyka et al. (72)*	22	21.40%	81	78.60%	NR	NR	NR	NR	NR	NR	11	21.60%	40	78.40%	NR
Tavel et al. (76)*	45	14%	285	86%	NR	51 (total number of females and males)	NR	NR	NR	NR	NR	NR	NR	NR	
Canales et al. (42)	5/19 (26%)	6%	82/220 (37%)	94%	*p* = 0.71	6/19 (32%)	11%	48/220 (22%)	89%	*p* = 0.32	NR	NR	NR	NR	NR
O’Connor et al. (47)	3/34 (%9)	15%	17/144(%12)	85%	NS	3/34 (%9)	9.40%	29/144 (%20)	90.60%	NS	NR	NR	NR	NR	NR
Eyer et al. (44)	13/221 (5.9%)	28%	33/606 (5.4%)	72%	NR	17/221 (7.7%)	28%	44/606 (7.2%)	72%	NR	NR	NR	NR	NR	NR
Isichei et al. (68)	8/49 (16%)	24%	25/153 (16%)	76%	NR	NR	NR	NR	NR	NR	6/49 (12%)	40%	9/153 (6%)	60%	NR
Salottolo et al. (54)	4/41 (9.7%)	14.81%	23/205 (11%)	85.19%	NR	NR	NR	NR	NR	NR	NR	NR	NR	NR	NR
Soyka et al. (73)	NR	NR	NR	NR	NR	46/325(14%)	34%	90/581 (15.5%)	66%	NS	NR	NR	NR	NR	NR
Ring et al. (71)^*^	26	17.30%	124	82.70%	NR	NR	NR	NR	NR	NR	NR	NR	NR	NR	NR
Soyka et al. (57)^*^	103 (total number of females and males, male predominance reported)				*p* < 0.01	151 (total number of females and males, male predominance reported)				*p* = 0.01	NR	NR	NR	NR	*p* < 0.001
Barrio et al. (64)	40/76 females (52.6%), 110/180 males (61.1%) with SAWS (seizures, disordered perceptions, or delirium)^a^, *p* value for sex difference in all complications = 0.2 26.7% females, 73.3% males^b^														
Foy et al. (58)	23/102 females (22.5%), 90/437 males (21%) with these complications (seizures, hallucinations, or delirium)^a^, no *p*-value 20.4% females, %76.6 males^b^														

* Studies started with patients who already presented with DT/seizures/hallucinations.

NR, Not reported.

NS, Non-significant.^a^Number of female or male patients with condition/total number of female or male patients (percentage).^b^Percentage of female or male patients with condition from the total number of patients with condition.

### 3.6. Sex difference in AWS treatment

Soyka et al. (57) reported sex differences in the side effects of combination therapy of tiapride and carbamazepine. Females exhibited more total side effects such as dyskinesia (*p* < 0.01), sedation, vertigo, somnolence (*p* < 0.05) and “others” (*p* < 0.01), while males suffered from more ataxia (*p* < 0.05) (57). Wojnar et al. (43) reported that compared to females, males received diazepam more frequently (males vs. females: 58% vs. 51% of episodes; *p* = 0.044) and at higher doses [a dose>30 mg administered: 9.5% (97/1016) vs. 3% (5/163), *p* = 0.04], but no sex differences in treatment with hydroxyzine or haloperidol was observed. In contrast, Canales et al. (42) observed that women received higher diazepam on hospital ward (Mean (SD) diazepam doses on hospital ward = 0.04 (0.1) vs. 0.12 (0.3) mg/kg, *p* = 0.01), but there was no significant sex difference in diazepam doses in the emergency department or in lorazepam doses neither in emergency department nor in the hospital ward. O’Connor et al. reported that the incidence of AWS treatment failure (such as prolonged withdrawal more than 5 days) was non-significantly higher in women compared to men [43% (62/145) vs. 53% (18/34), RR = 1.24, 95% CI:0.84–1.83] (47).

### 3.7. Sex difference in AWS mortality

In Wojnar’s study, 1% (*n* = 12) of AWS patients died during hospitalization and all of them were males. In total 19.5% (197/1,016) of males and 16% (26/163) of females died during the 16 years follow up period. Despite that the mean age at time of death was not significantly different between males and females hospitalized for AWS (males vs. females: 47.5 ± 10.8 vs. 48.8 ± 12.4 years, P = ns) (43), the time from first hospitalization for AWS to death was approximately 1.5 years shorter for men compared to women (3.9 ± 3.0 vs. 5.4 ± 3.3 years, *p* < 0.05). Campos et al. (2) reported that mortality risk tended to be higher for men [23.6% (256/1,085) vs. 18.3% (33/180), P = ns]; however, the effect disappeared after adjusting for smoking. In Monte et al.’s study (70), 6.38% of women (3/47) and 5.28% of men (26/492) with AWS died (mortality rate: 6.6, 95% CI: 4.2–9.1) (70). In Canales’s study, only a male MICU patient died (42) (Table 3).

**Table 3:** AWS mortality.

Study	Number (%) of deaths in female AWS patients	Number (%) of deaths in male AWS patients	*p*-value
Campos et al. (2)	33/180 (18.3%)	256/1,085 (23.6%)	NS
Canales et al. (42)	0/118 (0%)	1/1,378 (0.07%)	NR
Monte et al. (70)	3/47 (6.4%)	26/492 (5.3%)	0.7
Tavel et al. (76)	5/45 (11.1%)	34/285 (11.9%)	NR
Wojnar et al. (43)	26/163 (15.9%)	197/1,016 (19.3%)	NR

NR, Not reported.

NS, Non-significant.

## 4. Discussion

The results of this review highlight heterogenous methodology and inconsistent findings on potential clinical differences between men and women during AWS. Because of this heterogeneity, we were unable to conduct a meta-analysis. Despite hundreds of clinical studies on different aspects of alcohol withdrawal, several studies included only male patients (18, 19, 79, 80). Others included female patients, but the results were merged for both sexes (18, 81–84) making drawing a conclusion on sex difference impossible. While most of the literature on AWS focuses on treatment, the studies did not examine potential differences in treatment outcome between men and women (7, 13–17, 85–94). As such, our understanding and treatment of AWS in females follow the assumption that there are no sex differences in response to treatment, which may not be the case.

This dearth of sex-specific information is surprising given that a significant proportion of general hospital (8%) and ICU (20%) patients experience signs and symptoms of AWS (95), and still women were underrepresented in clinical studies. In our review, only 13% of all AWS patients were females. It is true that about a third of these studies were conducted before the year 2000, while the rapid increase in drinking among women has been witnessed over the past decade or two (20–23, 25–29). The observed increase in alcohol consumption rates, particularly among young women, could be related with menstrual cycle changes and associated distress (96–98). However, we did not include studies in our scoping review based on this variable, but rather on sex differences in alcohol withdrawal manifestations. Future studies are needed to highlight this aspect.

Our careful inspection of the current literature shows that while both men and women suffer from several medical and psychiatric comorbidities, extended hospital stay, high rates of ICU admission, complications during acute alcohol withdrawal, and high rates of mortality after discharge, there are several reported sex differences. Specifically, males appear to have higher rates of AWS-associated DT and mortality. Nonetheless, it is still a challenge to identify sex differences in many clinical aspects of AWS because of the heterogeneity of studies, small number of women included, and the inconsistent methodology. Large scale studies are urgently needed to examine potential differences between males and females in various aspects of the AWS.

### 4.1. Patients characteristics and risk factors

Two studies (43, 47) showed the time between alcohol misuse and first withdrawal of alcohol was shorter in women than men which supports telescoping phenomena. Telescoping refers to the pattern of females’ accelerated progression from the onset of alcohol drinking to alcohol-related problems and treatment seeking, when compared to males (47, 99). However, a more recent study suggested that the telescoping effect is not evident in the general population (48).

Several studies have found multiple factors that increase the risk and severity of AWS, including previous AWS episodes, higher drinking levels, concurrent illness, and abnormal clinical and laboratory findings (44, 81, 83, 84, 100, 101). When these risk factors are considered in terms of sex differences, the studies have revealed inconsistent results. Goodson et al. (100) concluded that sex was not a predictor of severe AWS development (DT and, or seizures); however, they included studies with predominantly male patients and also concluded that further research is needed to evaluate the effect of sex on the course of AWS. Similarly, Wood et al. (101) concluded in their review that having severe AWS was not more likely in men than in women [Likelihood ratio (LR) for men 1.3, 95% CI: 1.0–1.7]. Some studies report that male sex is a risk factor of developing severe AWS (40, 55), while others do not (46, 64).

### 4.2. Laboratory findings

Previous studies showed that thrombocytopenia (100, 102), low serum potassium, (100, 103), higher initial alanine aminotransferase (ALT) (100, 103), were observed among patients with severe AWS. In our review, Wojnar et al. (43) was the only study that gave laboratory results separately based on sexes and showed that anemia and low serum potassium level were observed more commonly among women, while increased ALT, and hypoproteinemia were observed among men. It is known that anemia is a more common finding in women (104). Likewise, a study evaluating comorbidities in AUD patients observed that women experience significantly more anemia (105). Therefore, it is unclear whether Wojnar’s finding of anemia more commonly in women with AWS simply reflects the more the finding across female population (43). Hypokalemia, reported by the literature as one of the most prominent risk factors for developing severe AWS was observed more frequently in women in Wojnar’s study. While total body potassium is lower in women, serum potassium concentration is sex-independent (106), therefore, the hypokalemia observed might be related with AWS. Male sex is related to higher serum ALT levels (107), and findings of higher ALT levels in male AWS patients (43) might be independent of AWS itself. However, as higher ALT levels were associated with severe AWS, higher ALT levels in males might put male patients at risk of developing more severe AWS. As such, it is possible to speculate that male patients with AUD and alcohol-associated liver disease are more likely to develop AWS, compared to females. Berggren et al. (65) reported that thrombocytopenia, a finding associated more commonly with severe AWS, was also observed more in male patients. However, it is known that men have lower platelet counts compared to women regardless of AWS (108), therefore observing thrombocytopenia more commonly in male AWS patients might not be related to AWS. As studies demonstrated that thrombocytopenia is a predictor of severe AWS (65, 100, 102), the normally lower platelet value in men can be interpreted as men may be more susceptible to developing severe AWS.

### 4.3. Medical and psychiatric comorbidities

Prior studies have shown that women are more sensitive to the toxic effects of alcohol (78, 109), have lower activity of alcohol dehydrogenase enzyme (110, 111), higher total body fat, less body weight and lower liver mass compared to men (5, 112). Therefore, women have higher blood alcohol concentrations for the same amount of alcohol than men (113). Women may be at higher risk of alcohol associated liver disease than men at any level of alcohol intake (114). Liver diseases and cirrhosis occur in women with shorter and lesser amounts of alcohol use compared to men (115). Two studies in our review support these findings (64, 78).

Wojnar et al. (43) reported that comorbid psychiatric disorders and substance use disorder were more common among males, while Schuckit et al. (39) concluded that there was no sex difference regarding these comorbidities. While Wojnar et al. retrospectively reviewed the records of patients hospitalized for AWS, Schuckit et al. conducted a structured interview with alcohol-dependent patients. Differences in methodology may explain, at least in part, these contrasting results. Also, the different regions and populations where the studies were conducted (Poland vs. USA) may also have confounded the results, as cultural drinking habits, availability of other addictive substances, and genetic factors may vary.

### 4.4. Hospital length of stay, ICU admissions

Wojnar et al. (43) reported that women needed longer course of hospital stay, while Canales et al. (42) reported that men needed longer hospital stay and ICU stay. The difference between the time periods (1997 vs. 2022) and the regions of the studies (USA vs. Europe) could contribute to the difference in findings. In addition, the difference in patients’ comorbidities may have contributed to these contradicting results, as Canales et al. (42) demonstrated that men experienced more sepsis and pneumonia. Ring et al. (71) did not observe sex difference regarding length of hospital stay; however, they found that comorbid pneumonia prolonged the hospital stay. As can be seen from this study, pneumonia itself might be the reason for longer hospital and ICU stay for males in Canales’s (42) study. Of note, women were older in Wojnar’s et al. (43) study population, while men were older in the Canales’s study. This difference in age across the two studies may be another factor that could have played a role in their different findings.

Overall the male to female ratio for all alcohol-related ICU admissions was 4.2:1 in the literature (116). Among the publications selected in our review, Canales et al. (42) was the only study that evaluated sex differences in ICU admissions in AWS and observed that the percentage of ICU admissions was similar (16%) for men and women. This finding can be interpreted as indicating that the severity of AWS does not differ between sexes, but more studies are needed to draw a solid conclusion.

### 4.5. AWS complications

One study (43) demonstrated that women were significantly more likely to develop DT, while most of the studies (37, 38, 40, 41, 57, 78) reported significantly higher number of males or no significant sex differences (42, 47) in DT cases. Two studies (43, 57) reported significantly higher number of AW seizures among males, while others (42, 73) did not find any significant difference. There were also studies (44–47, 64) that did not find sex differences in AWS complications. The studies that did provide sex-specific statistics on alcohol withdrawal seizures did not evaluate potential sex differences in phenotypes of alcohol withdrawal seizures and other seizure-related parameters such as seizure onset age, status epilepticus development, and treatment response. Similarly, studies did not evaluate characteristics of hallucinations in terms of sex difference. Male predominance seen in AWS complications might be related to underrepresentation of female participants or indeed male patients being more prone to complications of AWS. To understand this, studies examining the incidence of AWS complications in men and women separately are needed.

### 4.6. AWS treatment

Females were found to exhibit more treatment side effects (57), while there was a disagreement among studies regarding medication doses by sex (42, 43). These findings might be related to participant characteristics (comorbidities, age, severity of AWS). As with treatment of AUD, AWS treatment may require sex-specific individualization for optimal care (117). Due to the sparsity of literature on the topic, no reliable conclusion can be drawn on differences in management of AWS between men and women.

### 4.7. AWS mortality

Patients with AUD have increased mortality rates compared to the general population, and the mortality rate for men is greater than that for women (118). Similarly, Lewis et al. (41) found significantly shorter time from hospitalization to mortality among males with AUD compared to females. Moreover, men were found to have higher medical complications and more severe AUD than women. Consistent with these findings, one study (43) reported higher mortality rate and shorter time between first hospitalization and mortality in males, while two study did not report sex difference in AWS mortality (2, 70).

### 4.8. Limitations

The major limitation of our review of sex differences in AWS is the scarcity of research specifically examining this topic in a systematic manner. Most previous studies do not report separate data for males vs. females in all their results. In addition, even when reporting sex differences in results, studies reported predominance by comparing total number of males vs. total number of females for a particular outcome rather than comparing the sex-specific incidence of the outcome. The qualities of the studies were highly variable; as well, and not all studies reported *p*-values. In this review, not only were the studies heterogeneous based on geographical location, but also based on the treatment setting and populations. Studies focused on trauma patients (54, 55), emergency room patients (25, 37), inpatients who developed AWS (8, 25, 38, 44, 58, 59, 64, 68, 73–75, 77, 78), hospital patients presenting with AWS (39, 43, 45–47, 63, 67, 70, 71, 74), research center patients (39), psychiatric hospital patients (41), and detoxification unit patients (77). All study populations were mostly adults, however, only one study (75) focused on AWS in adolescents, which led to different results, as discussed previously.

### 4.9. Conclusion

Despite these limitations, our review highlights several significant differences in the clinical manifestations and treatment outcomes between men and women and brings more wariness to the unmet need to include more women in large-scale AWS studies and quantify sex specific differences and effects in the development, diagnosis, clinical manifestations and treatments of AWS and its complications. With the recent progressive increase in alcohol drinking among women, it is imperative for the scientific community to update the current research on AWS that has focused primarily on men and/or has not analyzed potential sex differences.

## Author contributions

HU: Conceptualization, Data curation, Formal Analysis, Investigation, Methodology, Project administration, Resources, Validation, Writing – original draft. MM: Investigation, Methodology, Writing – review & editing. HA: Methodology, Writing – review & editing. DK: Investigation, Methodology, Writing – review & editing. BA: Data curation, Supervision, Writing – review & editing. TS: Supervision, Writing – review & editing. LL: Supervision, Writing – review & editing. OA: Conceptualization, Data curation, Investigation, Methodology, Project administration, Resources, Supervision, Writing – review & editing.

## Funding

The author(s) declare financial support was received for the research, authorship, and/or publication of this article. This work was funded by the department of Psychiatry and Psychology at the Mayo Clinic Arizona (OAA). HU is supported by the Baskent University School of Medicine Hospital, Ankara, Turkey. LL is supported by NIDA/NIAAA, NIH IRP.

## Conflict of interest

The authors declare that the research was conducted in the absence of any commercial or financial relationships that could be construed as a potential conflict of interest.

The author(s) declared that they were an editorial board member of Frontiers, at the time of submission. This had no impact on the peer review process and the final decision.

## Publisher’s note

All claims expressed in this article are solely those of the authors and do not necessarily represent those of their affiliated organizations, or those of the publisher, the editors and the reviewers. Any product that may be evaluated in this article, or claim that may be made by its manufacturer, is not guaranteed or endorsed by the publisher.

## Supplementary material

The Supplementary material for this article can be found online at: https://www.frontiersin.org/articles/10.3389/fpsyt.2023.1266424/full#supplementary-material
