# Supplementary material for: Sex difference in alcohol withdrawal syndrome: a scoping review of clinical studies
Source: Front Psychiatry. 2023 Sep 22;14:1266424. doi: 10.3389/fpsyt.2023.1266424 (PMC10556532; doi:10.3389/fpsyt.2023.1266424)
Supplement: Supplementary file 1 [file Data_Sheet_1.docx]

**PubMed (304)** (((("alcohol withdrawal syndrome*") OR (("Substance Withdrawal Syndrome"[Mesh] AND alcohol) NOT (nicotine OR barbiturate* OR opioid*))) OR ("delirium tremens") OR ("Alcohol Withdrawal Seizures"[Mesh])) OR ("Alcohol Withdrawal Delirium"[Mesh])) AND ((("sex related difference*") OR ("sex specific")) OR ("sex difference*" OR "gender difference*" OR sex OR gender) OR ("Sex Characteristics"[Mesh])) Filters: English

**Embase (256)**

exp alcohol withdrawal syndrome/

exp delirium tremens/

exp alcohol withdrawal seizure/

exp alcoholic delirium/

exp *sex difference/

(gender adj1 difference*).mp. [mp=title, abstract, heading word, drug trade name, original title, device manufacturer, drug manufacturer, device trade name, keyword heading word, floating subheading word, candidate term word]

(sex or gender).mp. [mp=title, abstract, heading word, drug trade name, original title, device manufacturer, drug manufacturer, device trade name, keyword heading word, floating subheading word, candidate term word]

exp sexual characteristics/

5 or 6 or 7 or 8

(nicotine or barbiturate* or opioid*).mp. [mp=title, abstract, heading word, drug trade name, original title, device manufacturer, drug manufacturer, device trade name, keyword heading word, floating subheading word, candidate term word]

1 or 2 or 3 or 4

5 or 6 or 7 or 8

11 and 12

13 not 10

limit 14 to english language

<https://ovidsp.ovid.com/ovidweb.cgi?T=JS&NEWS=N&PAGE=main&SHAREDSEARCHID=4JlzkgFeLoH500xpvOuEQiHDNFgzUQ4PVVX1rQ0xs9eShSpbzNOnb1ORZ8t2SYyC>

**Scopus (173)** ( ( TITLE-ABS-KEY ( "alcohol withdrawal syndrome" OR "delirium tremens" OR "alcohol withdrawal seizure*" OR "alcohol delirium" ) ) AND ( TITLE-ABS-KEY ( "sex difference*" OR "gender difference*" OR sex OR gender OR "sex characteristic*" OR "sex related difference*" OR "sex specific" ) ) ) AND NOT ( ALL ( nicotine OR barbitruate* OR opioid* ) ) AND ( LIMIT-TO ( LANGUAGE , "English" ) )

**Web of Science (63)**

"alcohol withdrawal syndrome" OR "delirium tremens" OR "alcohol withdrawal seizure*" OR "alcohol delirium" (Topic) and "sex difference*" OR "gender difference*" OR sex OR gender OR "sex characteristic*" OR "sex related difference*" OR "sex specific" (Topic)

**AND**

"alcohol withdrawal syndrome" OR "delirium tremens" OR "alcohol withdrawal seizure*" OR "alcohol delirium" (All Fields) and "sex difference*" OR "gender difference*" OR sex OR gender OR "sex characteristic*" OR "sex related difference*" OR "sex specific" (All Fields)

**NOT**

nicotine OR barbitruate* OR opioid* (All Fields)

**AND**

**English** Languages

<https://www.webofscience.com/wos/woscc/summary/ce5adcf8-5b39-4535-81ec-df30677b92d0-6b7055d5/relevance/1>

**EBM Reviews - Cochrane Database of Systematic Reviews**

sex.ti. or sex.ab.

gender.ti. or gender.ab.

(delirium adj1 tremens).mp.

(alcohol adj1 withdrawal).ti. or (alcohol adj1 withdrawal).ab.

(nicotine or barbiturate* or opioid*).mp. [mp=title, abstract, full text, keywords, caption text]

alcohol.ti. or alcohol.ab.

3 and 6

4 or 7

1 or 2

8 and 9

<https://ovidsp.ovid.com/ovidweb.cgi?T=JS&NEWS=N&PAGE=main&SHAREDSEARCHID=5idajFx7BcclhwY4sxwTXjBVIvXmHxkenlOUagY1knBFAoZ7EYCPqIZ4brSBIYwly>

**Clinical trials.gov**

No Studies found for: "gender difference*" OR "sex difference*" | Alcohol Withdrawal

| DATABASE | RESULTS | DUPLICATES | REMAINING |
| --- | --- | --- | --- |
| PubMed | 304 | 0 | 304 |
| Embase | 256 | 53 | 203 |
| Scopus | 173 | 152 | 21 |
| Web of Science | 63 | 58 | 5 |
| Cochrane Database of Systematic Reviews | 0 | 0 | 0 |
| ClinicalTrials | 0 | 0 | 0 |
| **TOTAL** | 796 | 263 | 533 |

We did not use any automation tools for exclusion. We excluded 761 studies by a human.
