# Supplementary material for: Sex difference in alcohol withdrawal syndrome: a scoping review of clinical studies
Source: Front Psychiatry. 2023 Sep 22;14:1266424. doi: 10.3389/fpsyt.2023.1266424 (PMC10556532; doi:10.3389/fpsyt.2023.1266424)
Supplement: Supplementary file 2 [file Data_Sheet_2.docx]

Supplementary Items

Sex difference in alcohol withdrawal syndrome: a scoping review of clinical studies

Hayrunnisa Unlu, M.D. ^1,2^, Marie Michele Macaron, B.S.^3^, Hande Ayraler Taner, M.D.^2^, Duygu Kaba, M.D.^2,^ Burcu Akin Sari, M.D. ^2^, Terry D Schneekloth, M.D. ^1^, Lorenzo Leggio, M.D., Ph.D.^4^, and Osama A Abulseoud, M.D.*^1,5^

^1^Department of Psychiatry and Psychology, Mayo Clinic Arizona, Phoenix, Arizona, United States

^2^Department of Child and Adolescent Psychiatry, Baskent University School of Medicine Hospital, Ankara, Turkey

^3^St. Georges’ University of London, Medical School, United Kingdom

^4^ Section on Clinical Psychoneuroendocrinology and Neuropsychopharmacology, Translational Addiction Medicine Branch, National Institute on Drug Abuse, and National Institute on Alcohol Abuse and Alcoholism, NIH, United States

^5^Department of Neuroscience, Graduate School of Biomedical Sciences, Mayo Clinic College of Medicine, Phoenix, Arizona, United States

*Corresponding author

Osama A Abulseoud, M.D.

Department of Psychiatry and Psychology,

Mayo Clinic

5777 E Mayo Blvd, Phoenix, AZ 85054

Phone: (480) 342-2000

[Abulseoud.osama@Mayo.edu](mailto:Abulseoud.osama@Mayo.edu)

| Author, Year | Question 1 | Question 2 | Question 3 | Question 4 | Question 5 | Question 6 | Question  7 | Question 8 | Total | Quality |
| --- | --- | --- | --- | --- | --- | --- | --- | --- | --- | --- |
| Eyer et al., 2011 | * | * | * | NA | 0 | * | * | 0 | 5 stars |  |
| Foy et al,, 1997 | * | * | * | NA | * | * | * | 0 | 6 stars |  |
| Gómez- Méndez et al., 2018 | * | NA | * | NA | * | * | * | 0 | 5 stars |  |
| Himmels tein et al., 1938 | * | * | * | NA | 0 | * | * | 0 | 5 stars |  |
| Jarque- López et al., 2001 | * | * | * | NA | * | * | * | 0 | 5 stars |  |
| Lewis et al., 1995 | * | NA | * | NA | * | * | * | * | 6 stars |  |
| Marchan d et al., 2022 | * | * | * | NA | * | * | * | NA | 6 stars |  |
| Monte et al., 2009 | * | * | * | NA | * | * | * | NA | 6 stars |  |
| Monte et al., 2010 | * | * | * | NA | * | * | * | NA | 6 stars |  |
| Berggren et al., 2009 | * | * | * | NA | * | * | * | NA | 6 stars |  |
| Campos et al., 2011 | * | * | * | NA | * | * | * | NA | 6 stars |  |
| Patrick G. O’Connor et al., 1993 | * | NA | * | NA | * | * | * | 0 | 5 stars |  |
| Ring et al., 2021 | * | NA | * | NA | * | * | * | * | 6 stars |  |
| Salottolo et al., 2017 | * | * | * | NA | * | * | * | 0 | 6 stars |  |
| Schimmel et al., 2021 | * | * | 0 | NA | * | * | * | * | 6 stars |  |
| Sørensen et al., 2019 | * | * | 0 | NA | * | * | * | * | 6 stars |  |
| Soyka et al., 1988 | * | * | 0 | NA | * | * | * | NA | 5 stars |  |
| Soyka et al., 1989 | * | * | 0 | NA | * | * | * | NA | 5 stars |  |
| Soyka et al., 2006 | * | * | 0 | NA | * | * | * | * | 6 stars |  |
| Steel et al., 2021 | * | NA | * | NA | NA | * | * | * | 5 stars |  |
| Tavel et al., 1961 | * | NA | 0 | NA | NA | * | * | 0 | 3 stars |  |
| Wetterling & Junghanns, 2000 | * | NA | * | NA | * | * | * | 0 | 5 stars |  |
| Wojnar M. et al., 1997 | * | NA | * | NA | * | * | * | * | 6 stars |  |

Abbreviations:

*NA: Not Applicable*

**Supplementary Item 2:**

Newcastle-Ottawa Quality Assessment Form for Cohort Studies

Note: A study can be given a maximum of one star for each numbered item within the Selection and Outcome categories. A maximum of two stars can be given for Comparability.

Selection (maximum four stars)

1) Representativeness of the exposed cohort

a) Truly representative (one star)

b) Somewhat representative (one star)

c) Selected group

d) No description of the derivation of the cohort

2) Selection of the non-exposed cohort

a) Drawn from the same community as the exposed cohort (one star)

b) Drawn from a different source

c) No description of the derivation of the non-exposed cohort

3) Ascertainment of exposure

a) Secure record (e.g., surgical record) (one star)

b) Structured interview (one star)

c) Written self-report

d) No description

e) Other

4) Demonstration that outcome of interest was not present at start of study

a) Yes (one star)

b) No

Comparability (a maximum two stars)

1) Comparability of cohorts based on the design or analysis controlled for confounders

a) The study controls for age, sex and marital status (one star)

b) Study controls for other factors (one star)

c) Cohorts are not comparable based on the design or analysis controlled for confounders

Outcome (maximum three stars)

1) Assessment of outcome

a) Independent blind assessment (one star)

b) Record linkage (one star)

c) Self report

d) No description

e) Other

2) Was follow-up long enough for outcomes to occur

a) Yes (one star)

b) No

Indicate the median duration of follow-up and a brief rationale for the assessment above:

3) Adequacy of follow-up of cohorts

a) Complete follow up- all subject accounted for (one star)

b) Subjects lost to follow up unlikely to introduce bias- number lost less than or equal to 20% or description of those lost

suggested no different from those followed (one star)

c) Follow up rate less than 80% and no description of those lost

d) No statement

| Author, Year | Question 1 | Question 2 | Question 3 | Question 4 | Question 5 | Question 6 | Question  7 | Total | Quality |
| --- | --- | --- | --- | --- | --- | --- | --- | --- | --- |
| Nedic Erjavec et al., 2021 | * | * | 0 | ** | * | ** | * | 8  stars |  |
| Sanvisens et al., 2021 | * | * | 0 | ** | * | * | * | 7  stars |  |
| Schuckit et al., 1995 | * | * | 0 | * | * | * | * | 6 stars |  |
| Stewart & Brown et al., 1995 | * | * | 0 | ** | * | * | 0 | 6 stars |  |

Abbreviations:

*NA: Not Applicable*

NEWCASTLE - OTTAWA QUALITY ASSESSMENT SCALE

(adapted for cross sectional studies)

Selection: (Maximum five stars)

1) Representativeness of the cases:

a) Truly representative of the HCC patients (consecutive or random sampling of cases) (one star)

b) Somewhat representative of the average in the HCC patients (non-random sampling) (one star)

c) Selected demographic group of users.

d) No description of the sampling strategy.

2) Sample size:

a) Justified and satisfactory (≥ 400 HCC included) (one star)

b) Not justified (<400 HCC patients included)

3) Non-Response rate

a) The response rate is satisfactory (≥95%) (one star)

b) The response rate is unsatisfactory (<95%), or no description

4) Ascertainment of the screening/surveillance tool:

a) Validated screening/surveillance tool (2 stars)

b) Non-validated screening/surveillance tool, but the tool is available or described (one star)

c) No description of the measurement tool

Comparability: (Maximum one star)

1) The potential confounders were investigated by subgroup analysis or multivariable analysis.

a) The study investigates potential confounders (one star)

b) The study does not investigate potential confounders

Outcome: (Maximum three stars)

1) Assessment of the outcome:

a) Independent blind assessment (two stars)

b) Record linkage (two stars)

c) Self report (one star)

d) No description

2) Statistical test:

a) The statistical test used to analyze the data is clearly described and appropriate (one star)

b) The statistical test is not appropriate, not described or incomplete

| Author, Year | Question 1 | Question 2 | Question 3 | Question 4 | Question 5 | Question 6 | Question  7 | Question 8 | Question 9 | Question 10 | Total | Quality |
| --- | --- | --- | --- | --- | --- | --- | --- | --- | --- | --- | --- | --- |
| Barrio et al. | * | * | * | * | * | 0 | NA | * | * | * | 8 stars |  |
| Campos et al., 2011 | * | * | * | * | * | 0 | NA | * | * | * | 8 stars |  |
| Canales Jr et al., 2022 | * | * | NA | * | * | 0 | NA | * | * | * | 7 stars |  |
| Deshmuk h et al., 2003 | * | * | NA | * | * | 0 | NA | * | * | * | 7 stars |  |
| Martins et al., 2022 | * | * | * | * | * | * | NA | * | * | NA | 8 stars |  |
| Monte- Secades et al., 2017 | * | * | NA | * | * | * | NA | * | * | * | 8 stars |  |
| H.U. Isichei et al., 1994 | * | * | * | 0 | 0 | 0 | NA | * | * | * | 6  stars |  |
| Amaducci et al., 2021 | * | * | * | 0 | 0 | 0 | NA | * | * | NA | 6 stars |  |

Abbreviations:

*NA: Not Applicable*

Quality Assessment Criteria for Observational Studies, Based on the Newcastle-Ottawa Scale

Sample Selection (maximum six stars)

1) Representativeness of the exposed cohort

1. Truly representative of the average patient in the community (one star)
2. Somewhat representative of the average patient in the community (one star)
3. Selected group of users
4. No description of the derivation of the cohort

2) Selection of the non-exposed cohort

1. Drawn from the same community as the exposed cohort (one star)
2. Drawn from a different source
3. No description of the derivation of the non-exposed cohort

3) Ascertainment of exposure

1. Biological test (eg, blood/urine) (one star)
2. Structured interview (one star)
3. Written self-report that characterizes dose (current or cumulative) (one star)
4. Written self-report without quantification of exposure
5. No description

4) Precision of Exposure Dose Ascertainment

1. Amount and time (one star)
2. No information about amount and time

5) Ascertainment of exposure done prospectively or retrospectively

1. Prospectively (one star)
2. Retrospectively

6) Demonstration that outcome of interest was not present at start of study, OR baseline assessment

1. Yes (one star)
2. No

Comparability (maximum two stars)

1) Adjustment for confounding (rendering comparability of cohorts on the basis of the design or analysis)

1. Study accounts/controls for other substance use (one star)
2. Study controls for any additional factor (mental health comorbidity; medication use; severity of PTSD; mental health comorbidity and treatment; socioeconomic status) (one star)
3. No adjustment for potential confounders

Outcome (maximum three stars)

1) Assessment of outcome

1. Objective measure (one star)
2. Validated self-report measures (one star)
3. No information or non-validated measures

2) Was follow-up long enough for outcomes to occur?

1. Yes (need to define adequate follow-up period for outcome of interest) (one star)
2. No

3) Adequacy of follow-up of cohorts

1. Complete follow-up: all subjects accounted for (one star)
2. Subjects lost to follow-up unlikely to introduce bias; small number (less than 20 %) lost, or description was provided of those lost (one star)
3. Follow-up rate < 80% and no description of those lost
4. No statement
